# *Paenibacillus peoriae*: current knowledge and agricultural biotechnology potential of a close relative of *P. polymyxa*

**DOI:** 10.1007/s10482-025-02135-3

**Published:** 2025-07-23

**Authors:** Jakub Dobrzyński, Iryna Kulkova

**Affiliations:** Institute of Technology and Life Sciences-National Research Institute, Falenty, 3 Hrabska Avenue, 05-090 Raszyn, Poland

**Keywords:** Biocontrol, *Paenibacillus*, Spore-forming bacteria, Plant growth-promoting bacteria (PGPB), Sustainable economy

## Abstract

*Paenibacillus peoriae*, a member of the genus *Paenibacillus*, is a gram-positive, spore-forming bacterium closely related to *Paenibacillus polymyxa*. This species exhibits a wide range of metabolic capabilities, enabling it to thrive in diverse environments and produce bioactive compounds with potential applications in agriculture and biotechnology. Recent studies reveal its capacity to produce biocontrol agents, such as fusaricidins, polymyxins, and tridecaptins, along with hydrolytic enzymes that inhibit phytopathogens including *Fusarium*, *Rhizoctonia*, *Alternaria*, *Botrytis*, *Phytophthora* (*Oomycota* phylum). Additionally, this species was capable of directly promoting plant growth through various mechanisms, such as the production of indole-3-acetic acid (IAA), nitrogen fixation, phosphorus solubilization, and 1-aminocyclopropane-1-carboxylic acid (ACC) deaminase activity. *P. peoriae* strains also produce enzymes like cellulase and protease, essential for various industrial applications, and extracellular polysaccharides (EPS) demonstrating potential in bioremediation and heavy metal removal. Recent studies highlight its ability to synthesize 2,3-butanediol, a valuable industrial compound, further establishing its biotechnological significance. This review consolidates current knowledge on the genome, metabolites, and applications of *P. peoriae* while identifying research gaps and future directions for maximizing its potential in sustainable agriculture and biotechnology.

## Introduction

Bacteria of the genus *Paenibacillus* are gram positive or variable, mostly aerobic or relatively anaerobic, and are capable of spores which enables them to survive in adverse environmental conditions (McSpadden Gardener 2004; Grady et al. 2016). These bacteria are widely spread in various environments such as soil, water, plants, and even in extreme habitats. *Paenibacillus* spp. has a high metabolic diversity, which enables them to break down many complex organic compounds (Grady et al. 2016; Dobrzyński et al. 2023). Many species of this genus are capable of producing bioactive substances such as antibiotics (lipopeptides), phytohormones, enzymes (chitinase, glucanase, cellulase, nitrogenase) (Yuan et al. 2022a; Dobrzyński et al. 2023; Khodashenas Rudsari et al. 2024), and volatile organic compounds (VOCs) (Wu et al. 2020; Xie et al. 2024) making them valuable in agriculture as biocontrol agents for plant pathogens and direct plant growth stimulation (Li et al. 2022; Sun et al. 2022; Kulkova et al. 2024). Besides, metabolites of *Paenibacillu*s spp. may be used in the food, feed, textile and paper industries, where their metabolites can be used up to modify biopolymers and improve production process (Dobrzyński et al. 2023; Tinoco and Freire 2023).

*Paenibacillus* is a genus of bacteria in the phylum Firmicutes and is derived from the genus *Bacillus*. The split of the genus *Paenibacillus* (and a few others) came about on the basis of phylogenetic analysis resulting in five clusters. The species typical of the genus *Paenibacillus* is *Paenibacillus polymyxa* (formerly *Bacillus polymyxa*)—it became the first representative of the newly created genus in 1993 (Ash et al. 1994; Grady et al. 2016). *P. polymyxa* is the best studied and described representative of the genus *Paenibacillus*, as well as appearing to have the greatest potential for applications in agriculture or biotechnology (Ngashangva et al. 2022; Huang et al. 2024; Dobrzyński and Naziębło 2024). Nevertheless, it is worth noting *P. peoriae*, which several reports have shown is a close relative of *P. polymyxa* and may have a similar biochemical profile and thus commercial potential (Heyndrickx et al. 1996; Zhao et al. 2022). Therefore, the purpose of this mini-review is to summarize the current knowledge of the genome, metabolites, and application capabilities of *P. peoriae*, as well as to delineate gaps in knowledge about it and identify new lines of research whose development could contribute to a better use of *P. peoriae* in agriculture and biotechnology.

## Genome

As mentioned in the introduction, *P. peoriae* is a close relative of *P. polymyxa*. In the 1990s, for the first time on the basis of phylogenetic analyses, it was possible to delineate these two bacterial species. Montefusco et al. 1993 reclassified *P. polymyxa* (at that time *Bacillus*) strain LMG 11724 as *P*. *peoriae*. Later, authors Hendrix et al. (1996) analyzing three strains of *P. peoriae* proved great similarity to *P. polymyxa* and great variability in fatty acids between the three strains. The studied *P. peoriae* strains had an average G + C content of 45 to 47%. These values are also confirmed by recent studies. For instance, in *P. peoriae* HJ-2, the average G + C content was 45% (Jiang et al. 2022).

According to our best knowledge, one of the earliest or possibly the first draft genome of *P. peoriae* was produced in 2011 (Jeong et al. 2012). Researchers using CLC Genomics Workbench assembled 53 contigs of over 200 bp, with a maximum contig size of 902,703 bp; the genome size was determined to be 5.77 Mb. Jeong et al. (2012) observed that the strain they analyzed (*P. peoriae* KCTC 3763 T) showed the greatest similarity to *P. polymyxa* E681.

Recently, the previously mentioned Jiang et al. (2022) sequenced the genome of *P. peoriae* HJ-2 using the PacBio Sequel platform. The entire genome of the strain contained 6,001,192 bp, and they identified 5439 genes, including 5237 coding sequences, 39 rRNA genes, and 108 tRNA genes. Based on the Gene Ontology database, 2562 genes were classified into 27 functional groups, and based on the Clusters of Orthologous Groups of proteins (COG) annotation, 3608 genes were assigned to 24 COG categories (Jiang et al. 2022).

In turn, the whole genome of *P. peoriae* ZF390 contained 6,193,667 bp (chromosome—the average GC content 44.96%) with three plasmids, *pPlas1*, *pPlas2*, and *pPlas3* (Zhao et al. 2022). The authors identified a total of 5890 open reading frames (ORFs) and 5574 protein-coding genes (CDSs) in the strain and according to the COG database, 4001 functional genes were identified in the ZF390 genome, with most of the genes related to carbohydrate transport and metabolism, transcription, and general function prediction (Zhao et al. 2022). In the same year, Ngashangva et al. (2022)—using ABySS v.2.035—assembled the entire genome (de novo) of *P. peoriae* IBSD35, resulting in 66 contigs with a total length of 5,862,582 bp (GC content of 45.6%). In addition, the authors, using the NCBI Prokaryotic Genome Annotation Pipeline (PGAP), detected 5245 genes including 4983 coding sequences (CDSs) most of which, for example, are related to the environmental information processing and carbohydrate metabolism proteins.

The genomes of strains belonging to *P. peoriae* have found various key genes for biocontrol or direct promotion of plant growth. For instance Jeong et al. (2012) found several gene clusters in the genome responsible for the biosynthesis of antibiotics, including gene clusters for the biosynthesis of tridecaptin and a novel heptapeptide antibiotic, which was similar to fusaricidin, as well as other contigs that could potentially encode non-ribosomal peptide synthetase (NRPS). Interestingly, the researchers also identified genes encoding various enzymes, including cellulases, xylanases, amylases, and glucanases (Jeong et al. 2012). Similarly, in the genome of the *P. peoriae* ZF390 strain, 10 gene clusters associated with the synthesis of compounds were detected, including, for example, three clusters encoding NRPSs, type Ι polyketide synthases (T1PKS), trans-acyl transferase polyketide synthetases (TransAT-PKS), siderophore, and an NRPS-like protein, i.e. compounds which may be used in biocontrol of fungal and bacterial phytopathogens (Zhao et al. 2022). Additionally, authors using the Kyoto Encyclopedia of Genes and Genomes (KEGG) database suggested that key genes associated with biofilm formation may occur in the *P. peoriae* ZF390 genome (Zhao et al. 2022). Also, several key genes related to plant growth stimulation or environmental cycling were found in the genome of *P. peoriae* IBSD35. These genes included genes encoding nitrogenases—nifK and nifD and genes related to trehalose, cellobiose, and maltose (Ngashangva et al. 2022). Additionally, *P. peoriae* IBSD35 possessed 18 NRPS, including gene clusters associated with antimicrobial activity, for example trans-acyltransferase polyketide synthase (Trans AT-PKS), as well as butyrolactone, bacteriocin, lanthipeptide, NRPS-like, and lasso peptide (Ngashangva et al. 2022).

## Biologically active substances and their applications in agricultural biotechnology

### Biocontrol

Over the past decade, reports on the antimicrobial activity of *P. peoriae* strains have increased significantly. Numerous studies have demonstrated that *P. peoria*e exhibits broad-spectrum antagonism against various phytopathogens, including fungi, bacteria, and nematodes, employing diverse mechanisms such as the production of antimicrobial metabolites, stimulation of plant resistance, and secretion of hydrolytic enzymes (Liu et al. 2019; Olishevska et al. 2023; Booth et al. 2022; Araujo et al. 2020; Seldin 2011). Due to these multifaceted biocontrol properties, *P. peoriae* holds significant potential for agricultural biotechnology as a sustainable alternative to synthetic agrochemicals, reducing reliance on conventional chemical pesticides while promoting environmentally friendly crop protection strategies.

Strains of *P. peoriae* have demonstrated the ability to produce a wide range of antimicrobial compounds. These substances can inhibit the growth of plant pathogens either directly by disrupting fungal or bacterial metabolism or indirectly, by stimulating plant defense mechanisms such as systemic acquired resistance (SAR) and induced systemic resistance (ISR). To date, *P. peoriae* has been shown to produce several lipopetides. Strains of *P. peoriae* are well known for producing fusaricidins, such as fusaricidin A, B, C, D being among the most characteristic antimicrobial metabolites of this species (Zhao et al. 2022; Jiang et al. 2022; Yuan et al. 2022b; Dobrzyński and Naziębło 2024; Zheng et al. 2025). In addition to fusaricidins, *P. peoriae* has been shown to synthesize other NRPs, including polymyxins, tridecaptins, paenicidins, brevicidin, paenilate, paeninodin, pelgipeptin, paenilan, and octapeptins, further highlighting its considerable potential as a source of biologically active secondary metabolites (Ali et al. 2021; Jiang et al. 2022; Zhao et al. 2022; Olishevska et al. 2023). Thanks to the synthesis of these compounds, *P. peoria*e strains are capable of inhibiting the growth of various phytopathogens, including fungal species such as *Fusarium graminearum*, *Magnaporthe oryzae*, *Botrytis cinerea*, *Rhizoctonia solani*, and *Sclerotinia sclerotiorum*, as well as bacterial pathogens such as *Pectobacterium brasiliense* and *Xanthomona*s spp. For example, *P. peoriae* strain ZF390, by producing six metabolites including brevicidin, tridecaptin, paenilate, fusaricidin B, paeninodin, and octapeptin, was able to effectively inhibit the growth of *Pectobacterium brasiliense*, a soft rot-causing bacterium in cucumber plants (Zhao et al. 2022). Besides, *P. peoriae* strain ZBSF16 was shown by Yuan et al. (2022b) to possess gene clusters encoding fusaricidin B, polymyxin, tridecaptin, and paenicidin B, with these metabolites exhibiting antimicrobial properties that effectively protected grape leaves against ten fungal and two bacterial pathogens in vitro.

Furthermore, *P. peoriae* produces hydrolytic enzymes that can degrade fungal cell walls or stimulation of ISR, thereby enhancing its biocontrol efficacy. For instance, the fungistatic activity of strains RP20, RP51, and RP62 against *F. graminearum*, *Magnaporthe oryzae*, and *B. cinerea* has been attributed not only to fusaricidins (A, B, C, D) but also to the production of chitinase, protease, and β-1,3-glucanase (Ali et al. 2021). Also, in a recent in vitro study, Saadaoui et al. (2024) demonstrated that *P. peoriae* significantly inhibited the mycelial growth of *F. culmorum* and completely blocked macroconidia germination and sporulation, likely due to the production of enzymes such as cellulase. Moreover, it activates key defense pathways—cytokinin, salicylic acid (ISR), jasmonic acid (ISR), and shikimic acid—in wheat leaves and roots, collectively boosting pathogen resistance (Saadaoui et al. 2024).

Interestingly, Jiang et al. (2022) revealed genes involved in the synthesis of resistance inducers (*alsS, alsD, bdh*) and the flagellin gene (*flg*) in *P. peoriae* strain HJ-2. This flagellin was shown to induce intracellular signaling, activating plant defense mechanisms categorized as pathogen- or microbe-induced immunity (PTI/MTI). Importantly, the antifungal activity of *P. peoriae* HJ-2 was demonstrated on *Paris polyphylla* against *F. graminearum*, *F. solani*, *F. oxysporum*, *F. tricinctum*, and *F. concentricum* under both greenhouse and field conditions. Also, Araujo et al. (2020) conducted a study on the impact of *P. peoriae* on the rhizosphere microbiota of wheat roots in soil infected with *Pythium* sp. clade F, under both field and greenhouse conditions. Their findings revealed similar microbial successions in both environments, suggesting that *P. peoriae* consistently modulates the microbiota regardless of cultivation conditions.

There are also works showing the possibility of combining *P. peorie* with beneficial fungi. Zhu et al. (2024) demonstrated that combining the fungus *Trichoderma yunnanense* SR38 with the *P. peoriae* strain SR235 enhanced the resistance of *Crocus sativus* to tuber rot caused by *F. oxysporum*. This enhanced resistance was attributed to the binding of plant cell membrane receptors, the induction of local immunity, and the strengthening of the plant’s overall immune response. The effect was further corroborated by the accelerated synthesis of organic acids, including DL-3-phenylmaleic acid, 3-hydroxydecanoic acid, and (2S)-2-isopropylmalate (Zhu et al. 2024).

The remaining studies demonstrating fungistatic and bacteriostatic properties of *P. peoriae* are presented in Table 1.

**Table 1:** Biocontrol properties of *P. peoriae* strains

*P. peoriae* strains	Plant species	Experimental conditions	Effects	References
B-14372	Avocado tree *(Persea Americana)*	In vitro	Antifungal activity against *R. lauricola* (laurel wilt) as well as *F. euwallaceae* (Fusarium dieback)	Dunlap et al. (2017)
GXUN15128	*Arabidopsis thaliana*	In vitro	Antifungal activity against *R. solani* (damping-off, root rot, stem canker, sheath blight), *Colletotrichum musae* (banana Anthracnose), *Neofusicoccum parvum* (gumosis, cankers, dieback of trees), *Alternaria alternate* (leaf spots, early blight, fruit rot), *Plectosphaerella cucumerina* (root rot i crown rot), *Bipolaris sorokiniana* (spot blotch, root rot, crown rot), *Cryphonectria parasitica* (chestnut blight), *F. oxysporum* (Fusarium wilt), *Botryosphaeria dothidea* (cankers, dieback, fruit rot), and *Fusarium pseudograminearum* (Fusarium crown rot, head blight)	Wang et al. (2023)
RR8, RR12, RR33, RR34	Lettuce	In vivo, in vitro	Antifungal activity against *F. oxysporum* f. sp. *Lactucae* (Fusarium wilt of lettuce)	Yadav et al. (2021)
294	Pepper, cucumber	In vitro*, *in vivo	Antifungal activity against *Pythium ultimum* (damping-off of cucumber), *R. solani* (damping-off of pepper), *Phytophthora capsici* (Phytophthora blight, root rot, crown rot, fruit rot), *F. virguliforme* (sudden death syndrome (SDS))	Liu et al. (2017)
No strain name	*Pinus* species	In vivo	Antifungal activity against *Leptographium terebranti*s and *Grosmannia huntii,* the causal agents of blue stain and root diseases in Pinus species	Devkota et al. (2020)
MHJL1	Cotton	Pot experiment	Antifungal activity against *F. oxysporum* and *Verticillium dahliae (Verticillium wilt)*	Zheng et al. (2025)
YHR2-1	Tomato (*Solanum lycopersicum* L.)	In vitro	Antifungal activity against *F. oxysporum* f. sp. *lycopersici* races 1 and 2, *B. cenerea* (gray mold)	Roy et al. (2024)
RhAn32	Tomato (*Solanum lycopersicum* L.)	In vitro, *greenhouse*	Antifungal activity against *F. oxysporum* f. sp. *niveum*, *F. oxysporum* f. sp. *lycopersici* and *F. oxysporum* f. sp. *raphani*	Jung et al. (2012)
JC-3jx	*Dendrobium nobile*	In vitro	Antifungal and antimicrobial activity against *Pestalotiopsis* sp. (leaf spot, fruit rot, necrosis), *Phytophthora sojae (phytophthora root)*, *Phytophthora capsica* LT1534 (root rot, damping-off, fruit rot, and blight), *F. oxysporum* f. sp. *eleocharidis*, *Alternaria solani* (early blight), *Pyricularia oryzae* (rice blast disease), *Escherichia coli* CICC 10003(gastroenteritis, food poisoning), *Staphylococcus aureus* ATCC25923, and *Clostridium perfringens* ATCC13124	Li et al. (2024)
Pa86	Potato *(Solanum tuberosum L.)*	In vitro, in vivo	Antifungal activity against *R.solanacearum* (bacterial wilt)	Bahmani et al. (2021)

In summary, *P. peoriae* already appears to be a highly promising candidate for use as a plant protection agent. However, there are still very few field studies involving this bacterium that reflect the real-world conditions under which phytopathogens infect plants. Additionally, a more detailed understanding of the mechanisms underlying its biocontrol activity—particularly through analysis of plant gene responses involved in processes such as ISR—could significantly advance current knowledge and, indirectly, accelerate its commercialization in the agricultural biotechnology market.

### Direct plant growth promotion

Strains of *P. peoriae* are also capable of directly promoting plant growth, making them suitable for use as biofertilizers to reduce reliance on synthetic fertilizers. To date, research has documented that *P. peoriae* produces growth-promoting substances such as indole-3-acetic acid (IAA), 1-aminocyclopropane-1-carboxylate deaminase (ACC) deaminase, siderophores, solubilizes phosphorus (converting it from unavailable forms into plant-accessible ones) and fix nitrogen (Zhang et al. 2023; Roy et al. 2024, de Almeida Leite et al. 2024; Ma et al. 2024). For instance, inoculation of oat with *P. peoriae* LrM1—which produced IAA and ACC deaminase, fixed nitrogen, and solubilized phosphorus—enhanced root length and surface area under growth chamber conditions (Zhang et al. 2023). Interestingly, despite showing low phosphate solubilization potential in vitro, *P. peoriae* UFLA 03–10 was able to enhance growth (biomass) and nutrient accumulation in maize plants under greenhouse conditions (de Almeida Leite et al. 2024). This result may suggest that other plant growth-promoting (PGP) traits possessed by this strain were not detected in the study.

Moreover, there are also studies showing that a strain of *P. peoriae* can promote the growth of a plant classified as medicinal. In greenhouse experiments, *P. peoriae* SYbr421—a nitrogen-fixing and phosphorus-solubilizing bacterium—significantly increased the plant height, fresh leaf weight, and root biomass of *Dioscorea nipponica* (Dang et al. 2024).

In summary, by providing plants with accessible forms of nitrogen and phosphorus, *P. peoriae* can reduce the need for synthetic nitrogen and phosphorus fertilizers, which are known to contribute significantly to environmental degradation. Overuse of these fertilizers leads to nutrient runoff, causing eutrophication of water ecosystems, as well as increased emissions of nitrous oxide, a potent greenhouse gas (Wierzchowski et al. 2021; Górska et al. 2024). Therefore, integrating *P. peoriae*- based biofertilizers into agricultural systems offers a sustainable strategy for improving crop productivity while mitigating the ecological footprint of conventional fertilization practices.

## Other traits

In addition to its ability to produce substances involved in biocontrol and direct plant growth stimulation, *P. peoriae* can also produce other compounds that can be used for other branches of biotechnology. There are several strains of *P. peoria* that could potentially become a source of enzymes for various biotechnological processes. For instance Alvarez et al. (2006) found proteases in *P. peoriae* NRRL BD-62 that they believe can be used in various branches of biotechnology. The strain they tested showed the highest proteolytic activity after 96 h incubation, interestingly the activity was completely inhibited by 1,10-phenanthroline (zinc-metalloprotease inhibitor).

Moreover, cellulase was detected in *P. peoriae* MK1, which was subsequently cloned and expressed in *E. coli* (Kim et al. 2023). The pure enzyme showed the highest activity in the carboxymethyl cellulose (amorphous cellulose), which may indicate that it was an endoglucanase; this type of enzymes are widely used in industries such as paper and pulp (fiber modification), textiles (fabric softening and denim biostoning), food (juice clarification and fiber breakdown), bioenergy (biofuel production), agriculture (composting and soil enrichment), pharmaceuticals (drug processing), cosmetics (exfoliation), and environmental applications (waste recycling and bioremediation) (Dobrzyński et al. 2023).

One report also indicates that heavy metals can be removed with *P. peoriae.* Fella-Temzi et al. (2018) isolated a strain *P. peoriae* TS7 from the wheat rhizosphere that was able to remove lead through the production of extracellular polysaccharides (EPS). Such studies are promising for the potential application of *P. peoriae* in the bioremediation of lead-contaminated environments.

Interestingly, as mentioned in the previous section, the *P. peoriae* SYbr421 strain exhibited the highest efficiency in directly biotransforming *Dioscorea nipponica* tuber powder into diosgenin—a precursor of several hormones, including progesterone—highlighting its potential as a promising agent in medical biotechnology (Dang et al. 2024).

There are also several papers showing the ability of *P. peoriae* strains to produce 2,3-butanediol. It is an important industrial compound that is used to synthesize products such as high-value drugs, antiseptics, rubber, pest control agents, solvents, jet fuel, resins, paints, flavorings, humectants and antifreeze (Tinôco and Freire 2023). Bio-base production involving bacteria has great potential to reduce and over time replace the conventional chemical process to generate commercial interest products. The advantage of biological agents over chemical processes is that they require less energy, do not require expensive catalysts, and can be environmentally friendly on the basis of closed-loop economy principles and integrated biorefineries (Tinôco and Freire 2023). Tinoco et al. (2021) were the first to suggest the possibility of 2,3-butanediol production by *P. peoriae* (and other species of the *Paenibacillus* genus). Two years later, Tinôco and Freire (2023) were the first to achieve the production of 2,3-butanediol by a *P. peoriae* NRRL BD-62 in a pilot bioreactor. In the batch test, the researchers observed a productivity of 0.90 g/L/h and a yield of 0.40 g/g. Moreover, the implementation of fed-batch fermentation, nutrient control, and optimization of fermentation conditions significantly improved the productivity and selectivity of 2,3-butanediol production. (Tinôco et al. 2023a, 2023b). All tests produced results comparable to those achieved using unsafe and genetically modified microorganisms. Recent studies by Tinôco et al. (2024, 2025) have demonstrated that this strain exhibits high productivity and selectivity in the bio-based production of 2,3-butanediol under microaerobic conditions, utilizing a cost-effective NH₄Cl-based medium at a C/N ratio of 8.5 g/g without the need for external pH control. The breadth of these publications clearly underscores the significant economic and environmental potential of *P. peoriae*.

## Conclusions and future perspectives

**Fig. 1:**
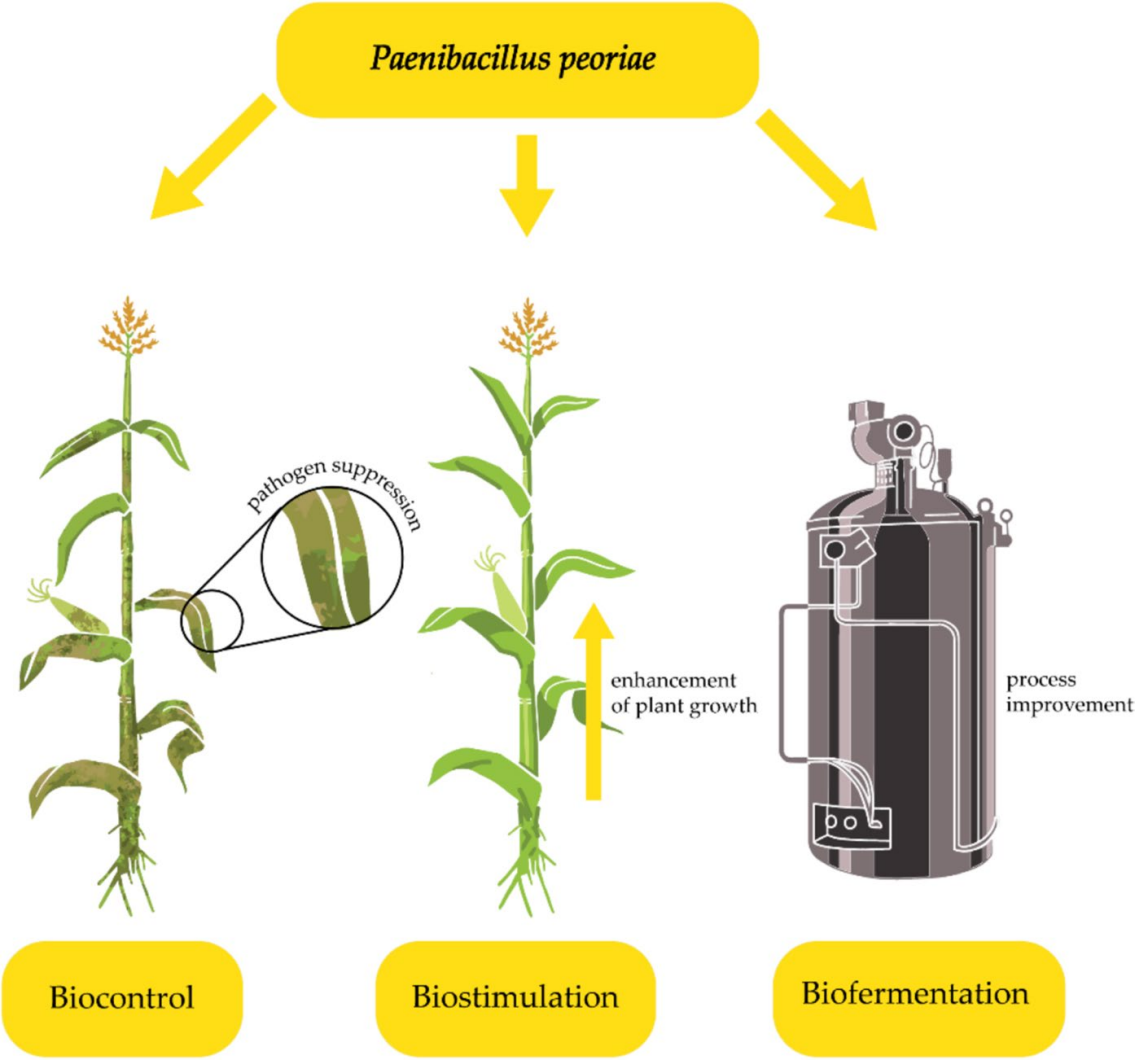
Main potential applications of *P. peoriae*

The growing interest in *P. peoriae* is due to its remarkable metabolic versatility and ability to produce a variety of bioactive compounds. Its biocontrol capabilities, driven by the production of antimicrobial peptides like fusaricidins and tridecaptins, hydrolytic enzymes, and siderophores, make it a valuable asset in sustainable agriculture. Beyond biocontrol, its potential as a plant growth promoter, enzyme producer, and biofermentation (bioconversion) agent further highlights its biotechnological promise (Fig. 1).

Notably, advancements in genome sequencing and functional annotation have identified genes and pathways responsible for these activities, paving the way for targeted applications.

Despite these advances, several research gaps remain. The mechanisms underlying the antimicrobial activities of *P. peoriae* require further elucidation, particularly in field conditions. Moreover, although bioactive substances are now known, we still have limited understanding of whether they act directly on the phytopathogen, ISR, or protect plants through other mechanisms. Importantly for *P. peoriae* and other strains, it is important to assess the effects on the native microbial community and its genes, which can also give information on the mechanism of action of the plant growth-promoting bacteria (PGPB) used (Dobrzyński et al. 2024a, b, 2025). Additionally, exploring its interactions with other beneficial microorganisms, such as fungi, could unlock synergistic effects in plant protection and growth.

Scaling up the production of metabolites, including 2,3-butanediol, and optimizing bioreactor conditions for industrial applications should be also prioritized. Furthermore, studies on its safety, environmental impact, and regulatory compliance are essential for broader commercialization.

In conclusion, *P. peoriae* represents a promising tool for sustainable agriculture and biotechnology. Future research should focus on bridging knowledge gaps, improving production processes, and developing integrated strategies for its application in real-world scenarios. By leveraging its full potential, *P. peoriae* could play a pivotal role in advancing sustainable and environmentally friendly solutions in agriculture, industry, and beyond.

## Data Availability

No datasets were generated or analysed during the current study.
